# An Argument for Temperance

**Published:** 1886-02

**Authors:** Tryon Edwards


### AN ARGUMENT FOR TEMPERANCE.

The anecdote is told of the celebrated Dr. Richardson, of London, that by a simple experiment he convinced an intelligent young man of the importance of total abstinence, when argument or appeal might have been in vain. The young man was singing the praises of the "ruddy bumpe" as he called it, and saying that it not only did him good, but that he could not get through the day without it.

Without attempting a direct reply, Dr. Richardson said, "will you be good enough to feel my pulse as I am standing here?"

He did so, and the doctor said, "Count it carefully, and tell me what it says."

"Your pulse," was the reply, beats seventy-four to the minute.'

The doctor then sat down in a chair, and asked him to count it again. He did so, and said, "it has gone down to seventy."

The doctor then laid himself down on the lounge, and said ". now count it again."

He did so, and exclaimed, "why, it is only sixty-four; what an extraordinary thing!"

The doctor then said, "when you lie down at night, that is the way Nature gives your heart rest. In sleep you know nothing about it, but the beating organ is resting to that extent; and if you reckon it up, you will see at once it is a great deal of rest, because in lying down the heart is doing ten strokes less every minute than before. Now multiply that by sixty and it is six hundred, and multiply that number again by the eight hours you may give to sleep, and, within a fraction it is five thousand strokes less than when you are awake. And as the heart throws out six ounces of blood at every stroke or pulsation, it makes a difference of thirty thousand ounces or nearly nineteen hundred pounds of lifting during the night or nearly eleven millions of ounces, or almost seven hundred thousand pounds of lifting in a single year; and this by so delicate an organ or instrument as the human heart. When I lie down at night without alcohol, that is the rest my heart gets. But when you take your wine or your whiskey, or grog of any kind, you do not get that rest, for the effect of alcohol or spirit is to increase the number of strokes. and instead of getting this rest, you put on something like fifteen thousand extra strokes, or some ninety thousand ounces of extra lifting in a single night; and the result is you rise up weak and exhausted, and unfit for the next day's work till you have taken another drink, which, in the end, increases the exhaustion, and rapidly wears away the life itself."

The young man acknowledged that all this was perfectly true, though it had never before struck him in that light. He carefully reckoned up the figures, and finding what it meant to be lifting up so many extra thousand ounces whenever he took a drink, he became a total abstainer, with every benefit, as he admits, to his pulse, his health, and his happiness.

Is not here a most striking and conclusive argument for teetotalism? Let every young man ponder it.—*Rev. Tryon Edwards, D.D.*

